# A Agriement Made and Enterd

**Published:** 1862-04

**Authors:** 


### A AGRIEMENT MADE AND ENTERD

*Into this the 15th november*

BETWEEN A. B. AND D. C. 1854

A. B agries to lern D C. to extract teeth insirt teeth on pivit and treat Scirvy and toothake destroyin the narve and clenseing the teeth how to make toothake tinktur and mouth wash and prepperiation for destroyen the narves of the teeth and all the necesaryes required in the above agremeant

A. B.

For the sed agriement D. C.

agriese to pay to A. B the som of $50.00.

D. C.

We give the above article of agreement as a model for such documents. It is given literally in every particular. The writer does not confine himself to the old method of orthography, nor indeed does he embrace the new, but strikes out in a channel of his own (we like originality.) The matter of punctuation he discards altogether. We would suggest to him a little closer attention to Webster and Wilson. T.

